# Hypothyroidism and rheumatoid arthritis: a two-sample Mendelian randomization study

**DOI:** 10.3389/fendo.2023.1179656

**Published:** 2023-05-30

**Authors:** Yang Gao, Zheng-Rui Fan, Fang-Yuan Shi

**Affiliations:** ^1^ School of Medical Technology, North Minzu University, Yinchuan, China; ^2^ Orthopedics Department, Tianjin Hospital, Tianjin, China; ^3^ School of Information Engineering, Ningxia University, Yinchuan, China; ^4^ Collaborative Innovation Center for Ningxia Big Data and Artificial Intelligence Co-founded by Ningxia Municipality and Ministry of Education, Ningxia University, Yinchuan, China

**Keywords:** two-sample Mendelian randomization, hypothyroidism, rheumatoid arthritis, variants analysis, causal relationship

## Abstract

**Background:**

Meta-analysis of genome-wide association studies (GWAS) data showed that the relationship between hypothyroidism and rheumatoid arthritis (RA) risk remains under debate. This study is conducted to test the causal relationship of hypothyroidism and RA.

**Methods:**

A two-sample Mendelian randomization (TSMR) analysis was employed to estimate the causality of hypothyroidism and rheumatoid arthritis in European ancestry and Asian ancestry. Integrating the effects generated by TSMR, functional annotations and noncoding variant prediction framework were applied to analyze and interpret the functional instrument variants (IVs).

**Results:**

The results of the inverse variance weighted method showed a strong significant causal relationship between hypothyroidism and risk of RA in European ancestry [odds ratio (OR) = 1.96; 95% confidence interval (CI) 1.49, 2.58; *p* < 0.001]. The outcomes of MR-Egger, weighted median, weighted mode, and simple mode also showed that hypothyroidism was significantly associated with increased risk of RA in European ancestry. The MR-PRESSO method also showed significant results [Outlier-corrected Causal Estimate = 0.70; standard error (SE) = 0.06; *p* < 0.001]. An independent dataset and an Asian ancestry dataset were applied to estimate and obtain the coincident results. Furthermore, we integrated the effect of variants in TSMR analysis, functional annotations, and prediction methods to pinpoint the single-nucleotide polymorphism (SNP) rs4409785 as one of the causal variants, which suggested that this variant could impact the binding of CTCF-cohesin and play a vital role in immune cells.

**Conclusion:**

In this study, we prove that hypothyroidism is significantly causally associated with increased RA risk, which has not been shown in previous studies. Furthermore, we pinpoint the potential causal variants in RA.

## Background

Rheumatoid arthritis (RA) is one of the most common autoimmune and chronic inflammatory diseases. Almost 0.5% to 1% of the world population are affected by RA, which leads to bone damage, disability, and premature mortality (1, 2). Both genetic and environmental triggers may contribute to the onset and progress of RA (3). Thyroid-related diseases including hypothyroidism and hyperthyroidism can be divided into subclinical stage and overt, and autoimmune thyroid disease can also occur in patients with other systemic autoimmune disorders such as RA (4–7). The association study of Graves’ disease patients with other autoimmune diseases showed that RA is one of the most frequently observed diseases (8). Graves’ disease is one of the causes of secondary hypophysitis and an autoimmune pattern such as lacking T regulator cells in hypophysitis is also seen with RA and other autoimmune conditions (9). A Mendelian randomization (MR) study found the causal relationship between Graves’ disease and RA (10).

Previous studies showed that the rate of thyroid dysfunction diseases, especially hypothyroidism, was significantly increased in RA patients, compared with the prevalence in controls (11). McCoy et al. reported that the different presence of hypothyroid disease between cohorts of patients with or without RA was not significant at the time of RA diagnosis in the earlier stage (12). Recently, the meta-analysis also showed that the risk of developing thyroid dysfunction particularly hypothyroidism was increased in RA patients (13). However, in many cases, the earlier diagnosed RA or hypothyroidism does not mean that it begins earlier; the relationship between hypothyroidism and RA remains elusive.

**Figure 1:**
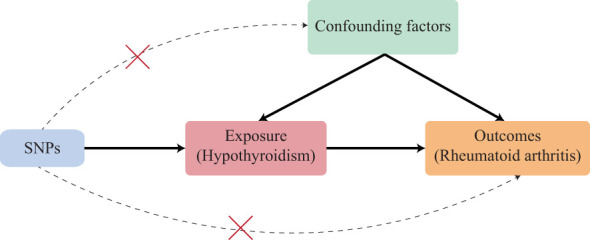
Schematic of an MR analysis. The causal estimation of hypothyroidism and RA. The first key assumption is that the genetic variant selected as the IVs should be significantly associated with exposure. The second key assumption is that the IVs should not be associated with any confounding factors. The third key assumption is that the IVs should affect the outcome merely through exposure but not any alternative pathways. SNP, single-nucleotide polymorphism.

MR uses the genetic markers as instruments of the exposure to assess the causal relationship between exposure and outcome (14). To decipher the causal relationship between hypothyroidism and RA, we adopted a two-sample Mendelian randomization (TSMR) that employed “MR-Egger”, “Weighted median”, “Inverse variance weighted”, “Simple mode”, and “Weighted mode” methods (**Figure 1**). Genome-wide association studies (GWAS) have identified a number of variants associated with diseases and traits that fall into noncoding regions (15). Though TSMR could infer the causal relationship between two diseases or traits, the causal variants in the instrument variants (IVs) are still hard to pinpoint. Furthermore, we also pinpointed the potential causal variants of IVs by integrating genomic and epigenetic annotations. Therefore, our study integrated MR and functional genomics data to investigate causal relationships between hypothyroidism and RA, and to pinpoint the potential causal variants to provide some clinical helpful insights for the diagnosis and therapy of RA patients.

## Methods

Five independent summary datasets were used to conduct MR analysis separately. The first summary data of RA were collected from the FinnGen R5 release (16), which included 6,236 cases and 147,221 controls of European ancestry in this dataset, and the IEU open GWAS project (ID: “finn-b-M13_RHEUMA”); the controls of the “M13_RHEUMA” were excluded from the listed items in M13_ARTHROPATHIES. The second summary data of RA were collected from IEU open GWAS project (ID: “ebi-a-GCST000679”), which included 5,539 cases and 20,169 controls of European ancestry. The RA cases in the second summary data of RA met the 1987 American College of Rheumatology (ACR) criteria for diagnosis of RA and were autoantibody-positive individuals; controls were matched to BRASS RA cases using principal components analysis from GWAS data from three separate studies (17). The hypothyroidism summary data were collected from the FinnGen R5 release (16) where hypothyroidism was defined as “hypothyroidism, drug reimbursement” (ID: “finn-b-HYPOTHY_REIMB”), meaning the phenotype of drug reimbursement of hypothyroidism patients, which included 7,183 cases, and 59,893 controls of European ancestry. The second hypothyroidism dataset defined as self-reported hypothyroidism/myxoedema was collected from IEU open GWAS project (ID: “ukb-a-77”), which included 16,373 cases and 320,783 controls in UK Biobank with filed Data-Field 20002 (https://biobank.ctsu.ox.ac.uk/crystal/field.cgi?id=20002). In addition, to analyze the treatment drug levothyroxine sodium relationship, we also used the hypothyroidism dataset with treatment/medication code: levothyroxine sodium summary data were collected from IEU open GWAS project (ID: “ukb-a-190”), which included 13,717 cases and 323,442 controls in UK Biobank with filed Data-Field 20003 (https://biobank.ctsu.ox.ac.uk/crystal/field.cgi?id=20003). The ancestry of the above datasets is European; findings were verified on the Asian ancestry using another independent dataset (18). GWAS summary statistics on RA with Asian ancestry were downloaded from the GWAS Catalog with 5,348 cases and 173,268 controls (http://ftp.ebi.ac.uk/pub/databases/gwas/summary_statistics/GCST90018001-GCST90019000/GCST90018690/harmonised/34594039-GCST90018690-EFO_0000685.h.tsv.gz). GWAS summary statistics on hypothyroidism with Asian ancestry was downloaded from the GWAS Catalog with 1,114 cases and 172,656 controls (http://ftp.ebi.ac.uk/pub/databases/gwas/summary_statistics/GCST90018001-GCST90019000/GCST90018642/harmonised/34594039-GCST90018642-EFO_0004705.h.tsv.gz).

To further verify the conclusion obtained from the above datasets, we adopted three recently larger datasets. We used the summary data of RA (finngen_R8_M13_RHEUMA, https://storage.googleapis.com/finngen-public-data-r8/summary_stats/finngen_R8_M13_RHEUMA.gz), which included 11,178 cases and 221,323 controls, and hypothyroidism summary data (finngen_R8_HYPOTHY_REIMB, https://storage.googleapis.com/finngen-public-data-r8/summary_stats/finngen_R8_HYPOTHY_REIMB.gz), which included 10,943 cases and 81,899 controls, collected from the FinnGen R8 release (19). In the second and third MR experiments, we adopted the summary data of RA in Okada et al., which included 14,361 cases and 42,923 controls of European ancestry (http://plaza.umin.ac.jp/~yokada/datasource/files/GWASMetaResults/RA_GWASmeta_European_v2.txt.gz); the cases in this dataset fulfilled the 1987 criteria of the ACR for RA diagnosis or were diagnosed as RA by professional rheumatologists (20). The cases in our study from FinnGen and UK Biobank were the registry data with clinically meaningful disease endpoints by the clinical expert groups.

### Instrument variants selection

Variants related to hypothyroidism and RA risk at *p*-value < 5×10^-8^ were identified from GWAS summary data of European ancestry. To obtain the independent IVs, we used the 1,000 Genome linkage disequilibrium (LD) in European ancestry with *r* ^2^ larger than 0.01 in the 5,000-kb region as the threshold to apply data clumping. For the outcome variants, the minimum *r* ^2^ is 0.8 and the palindromic single-nucleotide polymorphisms (SNPs) are allowed. We also used the minor allele frequency (MAF) threshold 0.3 to infer palindromic SNPs. *F* statistic was employed to evaluate the impact of IVs (21), the formula is *R* ^2^×(*N* − 2)/(1 − *R* ^2^), where *R* ^2^ is the cumulative explained variance of selected SNPs in exposure that used (2×EAF×(1 − EAF)×beta^2^)/[(2×EAF×(1 − EAF) ×beta^2^) + (2×EAF×(1 − EAF)×*N*×SE(beta)^2^)], where *N* is the sample size of research, EAF is the effect allele frequency, beta is the estimated genetic effect, and SE (beta) is the standard error of the beta. The strong IVs were chosen when *F* > 10.

### MR analysis

The three key assumptions (22) of IVs that are vital to the valid IVs for causal inference in MR analysis should be satisfied (1): the selected IVs should be directly associated with exposure; (2) the selected IVs should not be associated with confounders; (3) the selected IVs must have no effects on the outcome other than through the exposure (no horizontal pleiotropy exists).

Five complementary methods of TSMR were conducted on GWAS summary data to estimate the causal relationship that included MR-Egger, weighted median, inverse variance weighted (IVW), simple mode, and weighted mode. The IVW method, which uses the ratio estimate of each variant in a fixed effect meta‐analysis to estimate the causal effect (23), is strictly based on the three assumptions. Under the INSIDE assumption (instrument strength independent of the direct effects) (22), the MR-Egger method conducts a weighted linear regression rather than setting the intercept to be zero in IVW, and the intercept in MR-Egger can be used to estimate the horizontal average pleiotropic effect (24). If up to 50% of genetic variants are invalid, the total weight of the instrument comes from the median of the weighted ratio estimates of valid variants, then this method is called the weighted median method (25). We also incorporated mode-based methods that include simple mode and weighted mode. These methods estimate the causal effect of individual SNPs to form clusters (26). The simple mode takes the largest cluster of SNPs’ causal estimation, but the weighted mode assigns the weights to each SNP (27). If the beta values in summary data of exposure and outcome have significantly different distributions, the correct factor will be used.

The TwoSampleMR package (version 0.5.6) in R (version 4.0.2) was used to carry out the five MR methods.

### Pleiotropy, heterogeneity, and sensitivity evaluation

We required the directions of all five methods to be consistent, and *p*-values less than 0.05 indicate significant results. The MR-Egger method was performed to return intercept values to test the horizontal pleiotropy. If directional pleiotropy was detected with the *p*-value < 0.05, we employed MR-PRESSO to evaluate the horizontal pleiotropy in the generated MR model and removed the outlier and then estimated the causal effect again. Cochran’s *Q* statistic of IVW was applied to check the effect of heterogeneity. Leave-one-out and single SNP analyses were also performed to identify if a single SNP is driving the causal estimates.

### Functional instrument variant prioritization

We merged all IVs and used the b values in single SNPs analysis as the b score of each variant. We extracted variant call format (VCF) files of the merged IVs and adopted the Sei framework (28) (https://github.com/FunctionLab/sei-framework), which integrated 21,907 chromatin profiles to predict functional noncoding variants. We used the max absolute difference in 40 sequence classes of each variant as the Sei score. The top 10 variants with high b and Sei scores were the selected variants to reveal the potentially affected features in a more detail manner. The framework of functional analysis with details is described in **Supplementary Figure 1**. To visualize the regulatory features of selected variants in immune cells, we downloaded ReMap (https://remap.univ-amu.fr/storage/remap2022/hg38/MACS2/remap2022_all_macs2_hg38_v1_0.bed.gz) and CCCTC-binding factor (CTCF), estrogen receptor alpha gene (ESR1), androgen receptor (AR), and recombinant forkhead box protein A1 (FOXA1) chromatin immunoprecipitation sequencing (ChIP-seq) data (**Supplementary Table 1**). GWAS Catalog track was downloaded from the UCSC genome browser (https://genome.ucsc.edu/cgi-bin/hgTables?hgsid=1509267435_AXk036LRJbjcQTT5fpxOODCrVW6Y). PolyPhen-2 (29) was used to predict the function of variants falling into coding regions.

## Results

### RA and hypothyroidism, drug reimbursement

The results showed a significant causal relationship between hypothyroidism, drug reimbursements of hypothyroidism and risk of RA in the European ancestry through the IVW method (OR = 1.96; 95% CI 1.49, 2.58; *p* < 0.001). The result of the MR-Egger method (OR = 3.08; 95% CI 1.57, 6.04; *p* = 0.006) also showed a significant result.

**Figure 2:**
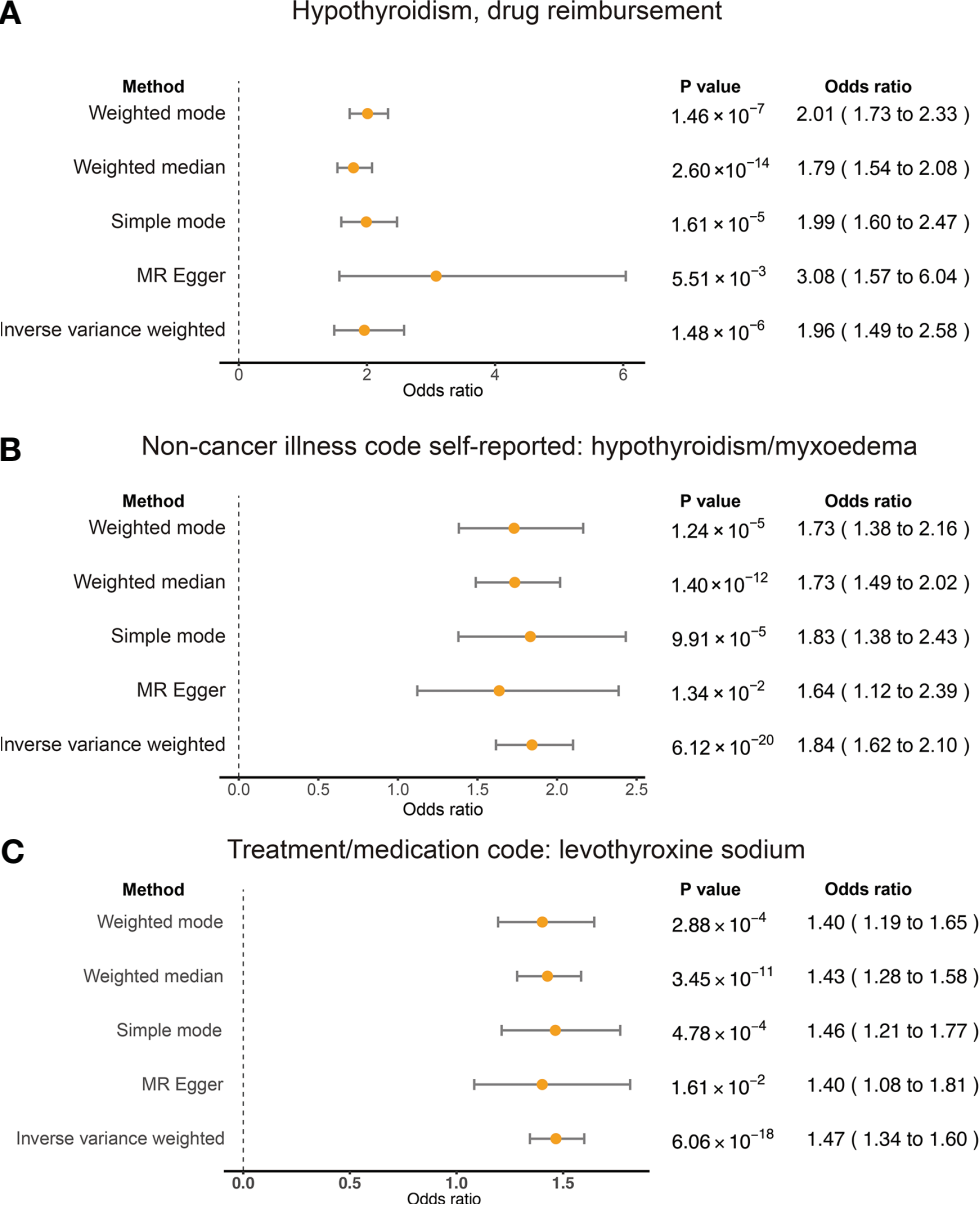
Summary view of the MR results. Summary of MR analysis results derived from the inverse variance weighted, MR-Egger, simple mode, weighted mode, and weighted mean methods. RA was used as the outcome. The hypothyroidism and drug reimbursements **(A)**, non-cancer illness code self-reported: hypothyroidism/myxoedema **(B)**, and levothyroxine sodium **(C)** as the exposures and significant associations were detected for these traits and RA risk.

**Figure 3:**
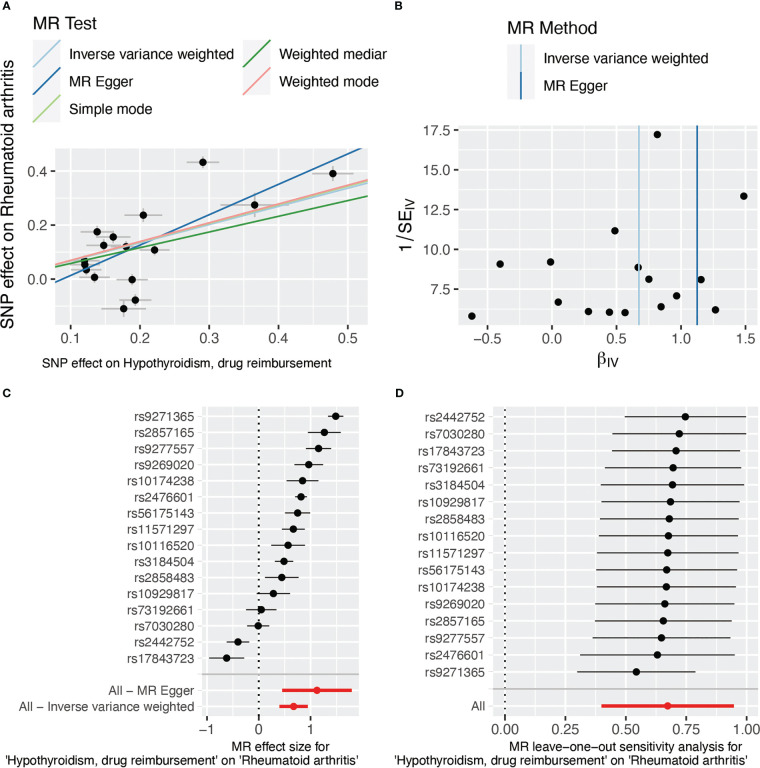
SNP effect evaluation in TSMR between hypothyroidism, drug reimbursement, and RA. **(A)** MR test scatter plot of five methods. The *x*-axis is the SNP effect on hypothyroidism, drug reimbursement. The *y*-axis is the SNP effect on rheumatoid arthritis. **(B)** MR funnel plot of IVW and MR-Egger methods. **(C)** Forest plot of MR sensitivity analysis. All MR-Egger and IVM methods showed that MR effect sizes that are larger than 0 mean that hypothyroidism, drug reimbursement had a causal effect on RA. **(D)** Forest plot of MR leave-one-out sensitivity analysis. MR, Mendelian randomization; SNP, single-nucleotide polymorphism.

The results of weighted median, simple mode, and weighted mode also showed a significant relationship between hypothyroidism, drug reimbursements of hypothyroidism and risk of RA (**Figure 2A**). MR-Egger (Cochran’s *Q* = 331.25; *p* < 0.001) and IVW methods (Cochran’s *Q* = 379.72; *p* < 0.001) were used to analyze the heterogeneity of outcomes, and there was heterogeneity among the selected IVs. Then, the IVW (multiplicative random effects) method was adopted to estimate the causal relationship, and the consequence was consistent (OR = 1.96; 95% CI 1.49, 2.58; *p* < 0.001). Though the heterogeneity existed among the selected IVs, it does not affect the results of IVW, and the relationship between hypothyroidism, drug reimbursements of hypothyroidism and risk of RA is still significant. MR-Egger was used to evaluate horizontal pleiotropy among the selected IVs; the results (β intercept = −0.09; SE = 0.07; *p* = 0.174) showed that no pleiotropy would affect the results (**Figure 3**). The MR-PRESSO method also showed significant results (Outlier-corrected Causal Estimate = 0.70; SE = 0.06; *p* < 0.001).

### RA and self-reported hypothyroidism/myxoedema

To test the conclusion from hypothyroidism, drug reimbursements of hypothyroidism and risk of RA, we also applied TSMR on self-reported hypothyroidism/myxoedema and RA of European ancestry. Five methods all showed significant relationships between self-reported hypothyroidism/myxoedema and RA of European ancestry. The IVW result showed that self-reported hypothyroidism/myxoedema significantly increased the risk of RA (OR = 1.84; 95% CI 1.62, 2.10; *p* < 0.001). MR-Egger also showed a significant relationship between the exposure and outcome (OR = 1.64; 95% CI 1.12, 2.39; *p* = 0.013) (**Figure 2B**). The heterogeneity results evaluated by MR-Egger (Cochran’s *Q* = 84.10; *p* = 0.005) and IVW (Cochran’s *Q* = 84.10; *p* = 0.007) showed that there was heterogeneity among the selected IVs. MR-Egger was used to evaluate horizontal pleiotropy among the selected IVs, and the results (β intercept = 5×10^-4^; SE = 0.01; *p* = 0.970) showed that no pleiotropy would affect the results. In addition, the results of single SNP analysis and leave-one-out sensitivity analysis indicated that no single SNP significantly leads to the causal results (**Supplementary Figures 2C, D**).

### RA and levothyroxine sodium

Levothyroxine sodium is a medicine used to treat hypothyroidism (30). We also found in the trait of people who took levothyroxine sodium, which had significant causal effects on RA risk. Forty-two SNPs were selected as the IVs and we used MR-Egger to analyze the horizontal pleiotropy; we further found that there was pleiotropy among IVs (β intercept = −0.07; SE = 0.04; *p* = 0.060). We then adopted MR-PRESSO to analyze and filter the nine outlier SNPs, and used MR-Egger again to test the horizontal pleiotropy and eliminate the horizontal pleiotropy (β intercept = 0.02; SE = 0.02; *p* = 0.245). The causal relationship was evaluated with the selected IVs; the IVW method (OR = 1.47; 95% CI 1.36, 1.60; *p* < 0.001) and the MR-Egger method (OR = 1.40; 95% CI 1.08, 1.81; *p* = 0.001) also showed significant consequence (**Figure 2C**). MR-Egger (Cochran’s *Q* = 31.43; *p* < 0.001) and IVW methods (Cochran’s *Q* = 33.14; *p* < 0.001) were used to analyze the heterogeneity of outcomes, and there was heterogeneity among the selected IVs. The IVW (multiplicative random effects) method (OR = 1.49; 95% CI 1.37, 1.62; *p* < 0.001) also showed significant results. Moreover, the single SNP analysis and leave-one-out analysis showed that no single SNP affects the causal result significantly (**Supplementary Figures 3C, D**).

### Functional analysis of IVs

**Figure 4:**
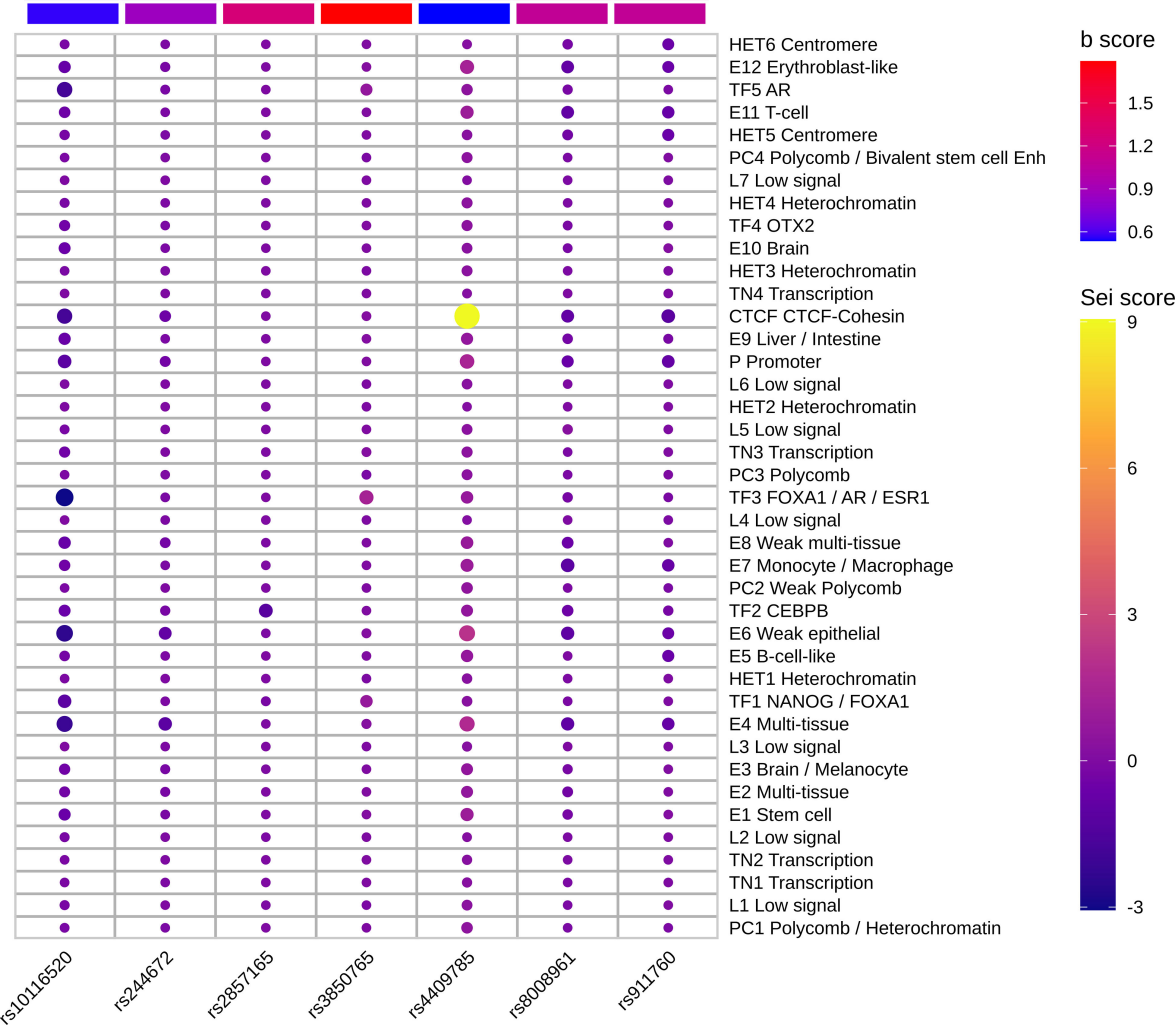
Top functional IVs. Heatmap of top 7 functional IVs. The Sei score is the fold change of each variant in 40 sequence classes. The b score is the b value in the sensitivity analysis of each variant. The SNP rs4409785 has the largest Sei score and b score > 0.5. The SNP rs3850765 has the largest b score and absolute Sei score > 1.

**Figure 5:**
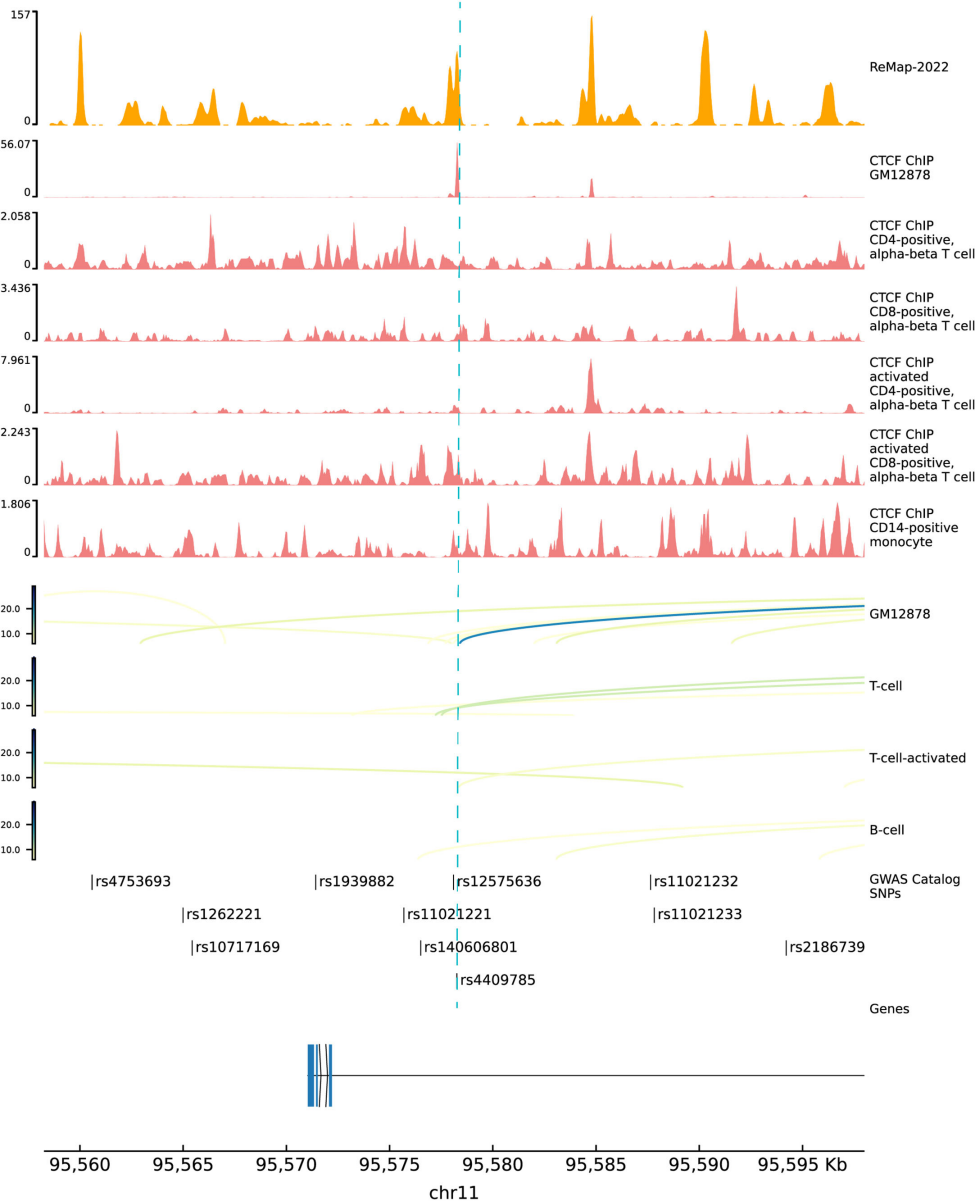
Functional annotations of SNP rs4409785. The top 1 track is the density track of ReMap 2022. The top 2 to 7 tracks are CTCF ChIP-seq bigwig peaks in immune cells. The top 8 to 11 tracks are ChIA-pet data in GM12878, T cell, activated T cell, and B cell. ChIP-seq, chromatin immunoprecipitation sequencing; ChIA-pet, chromatin interaction analysis by paired-end tag sequencing.

We deciphered the relationship between hypothyroidism and RA, but MR results could not provide the potential functional mechanisms of these diseases. Previous studies showed that almost 90% of variants in GWAS fall into noncoding regions. Based on the third assumption of MR analysis, the selected IVs have to affect the outcome through exposure. Selected IVs of three experiments were merged into a 102-variant dataset; Chen et al. developed the Sei framework (28) to discover the regulatory code of human genetics data based on sequence information of traits and diseases. We adopted Sei framework to predict the 92 variants and regarded the max absolute fold change in the 40 sequence classes (**Supplementary Table 2**). To obtain the MR estimates using each of the selected IVs singly, we employed the Wald ratio to perform the single SNP MR analysis. We used the b values in the single SNP analysis as b score. Integrating b score and Sei score, we filtered the top 7 variants with absolute Sei score > 1 and b core > 0.5 (**Figure 4**). SNP rs3850765 has the largest b score and a larger fold change on the TF3 sequence class such as FOXA1, AR, and ESR1 (**Supplementary Figure 4**). The UCSC genome browser annotation suggested that this variant fell into the regulatory regions (**Supplementary Figure 5**). Another SNP rs4409785 has the largest fold change on CTCF and CTCF-cohesin and a larger b score in self-reported hypothyroidism/myxoedema and RA, and levothyroxine sodium and RA. This variant fell into the regulatory enrichment regions and the peak of CTCF in GM12878. Compared with the CD8-positive alpha-beta T cell, the variant locus is more activated in the activated CD8-positive alpha-beta T cell in CTCF-ChIP data. The chromatin interaction analysis by paired-end tag sequencing (ChIA-PET) of CTCF in GM12878 also showed this variant located at the anchor region (**Figure 5**). The UCSC genome browser annotations showed that this variant fell in regulatory regions (**Supplementary Figure 6**).

## Discussion

In this study, two independent MR analyses were conducted by using European ancestry GWAS data to detect the relationship between hypothyroidism and RA risk, and found that hypothyroidism has a causal effect on RA risk. Moreover, because levothyroxine sodium is used to treat hypothyroidism, we also found that the genetic data of people who have hypothyroidism have a causal effect on RA risk. To validate the conclusion, we adopted larger datasets. Based on the FinnGen R8 release, the relationship of hypothyroidism, drug reimbursements between hypothyroidism and risk of RA in European ancestry is significant with the IVW method (OR = 1.98; 95% CI 1.34, 2.92; *p* < 0.001) (**Supplementary Figure 7A**). The results of Non-cancer illness code self-reported: hypothyroidism/myxoedema (OR = 2.13; 95% CI 1.52, 2.97; *p* < 0.001) and Treatment/medication code: levothyroxine sodium (OR = 24.92; 95% CI 7.05, 88.15; *p* < 0.001) with RA in the larger dataset also showed a significant relationship between these phenotype (**Supplementary Figures 7B, C**). The results of the other four methods also suggested a significant relationship, which validated the conclusion in the main datasets. To investigate whether the conclusion of hypothyroidism and RA reached from those with European ancestry also applies to Asians, a replication TSMR study using Asian GWAS data was also conducted. Though rs1049087 (*F* statistic = 30. 08) is the only significant lead SNP in the GWAS of hypothyroidism after filtering by conditions, we used the Wald ratio method but still obtained the same conclusion (OR = 2.12; 95% CI 1.79, 2.51; *p* < 0.001).

A reverse direction of hypothyroidism and RA TSMR analysis showed that there was no significant causal relationship between RA and hypothyroidism with the MR-Egger method. However, the IVW method showed a significant causal relationship between RA and hypothyroidism with no significant horizontal pleiotropic effect (**Supplementary Table 3**). Previous studies have reported that patients with RA are at high increased risk of thyroid dysfunctions (31). However, the earlier diagnosis does not mean which one starts earlier. Nisihara et al. (32) reported that rheumatic autoantibodies have been detected from 17.5% of autoimmune thyroid disease patients without clinical rheumatic disorder for 5 years. It suggested the need to closely monitor hypothyroidism patients so that RA can be diagnosed early.

MR is now widely used to assess the causal relationship through genetic markers between exposure and outcome (33). For the coding variants of IVs, we adopted PolyPhen-2 to predict the effect. SNP rs3775291 was predicted to be "likely damaged", which fell into the gene toll-like receptor 3 (*TLR3*) to change the amino acid leucine to phenylalanine. *TLR3* is highly expressed in the thyrocytes of Hashimoto’s disease patients and may affect the innate immune response (34). Moreover, toll-like receptors play complex roles in the pathogenesis of RA; arthritis was ameliorated when interference targeted *TLR3* in rats (35), which suggested the important role of *TLR3* in both thyroid disease and RA. Though the genetic markers always come from GWAS data, the functional molecular mechanism remained elusive since 90% of disease-related GWAS SNPs fall into the noncoding regions. The complex regulatory code in noncoding regions made it difficult to decipher the key SNPs and mechanisms. We found that the SNP rs4409785 locus is more activated in the activated CD8-positive alpha-beta T cell in CTCF-ChIP data. CTCF transcription factor binding sites are often found next to the binding sites of thyroid hormone receptors (36). Studies in human samples suggested that the CD8-positive T cell plays an important role in the initiation and maintenance of the disease process (37). A new strategy was proposed here; we mined the causal effects deeper in IVs. In our study, we integrated the effects of IVs evaluated by MR analysis, noncoding variant predicted tool, and functional genomic annotations to pinpoint the potential functional noncoding variants in hypothyroidism and RA risk.

Although we obtained significant results from the TSMR methods, there were still several limitations that should be noted. First of all, the traits of hypothyroidism are derived from the public GWAS dataset with hypothyroidism, drug reimbursement, and self-reported hypothyroidism with the non-cancer illness code, rather than the diagnosis code; the more specific diagnosis code of the GWAS dataset of hypothyroidism will increase the statistical power. Second, the number of GWAS data of hypothyroidism, especially the case number, is small. The statistical power will be increased with GWAS data with a larger sample size. Third, it has been reported that women and elderly RA patients have an increased risk of developing hypothyroidism (31), but the GWAS summary data in our study were unable to adjust for gender and age because the information was not available. Finally, the result from MR reflects the change in RA risk due to a lifelong change in exposure hypothyroidism status, rather than a specific time in life (38). Therefore, the short-term effect size from MR analysis merits additional investigation and should not be regarded as a short-term intervention.

## Conclusions

In summary, in this study, we provided strong evidence of hypothyroidism having a causal effect on RA risk. Importantly, we provided the potential critical SNPs in IVs and biological functional genomics annotations of these variants. Although translating findings into treatment required further validation, we still provided evidence that hypothyroidism patients need to be monitored for RA risk, which might help in the early detection of RA.

## Data availability statement

The original contributions presented in the study are included in the article/**Supplementary Material**. All code is available on Github (https://github.com/shify-nxu/MR-RA). Further inquiries can be directed to the corresponding author.

## Author contributions

YG: Methodology, software, data curation, formal analysis, visualization, writing—original draft, and writing—review and editing. Z-RF: Data curation, formal analysis, and writing—review and editing. F-YS: conceptualization, project administration, supervision, funding acquisition, resources, and writing—review and editing. All authors contributed to the article and approved the submitted version.
