# Supplementary material for: Hypothyroidism and rheumatoid arthritis: a two-sample Mendelian randomization study
Source: Front Endocrinol (Lausanne). 2023 May 30;14:1179656. doi: 10.3389/fendo.2023.1179656 (PMC10262846; doi:10.3389/fendo.2023.1179656)
Supplement: Supplementary file 1 [file DataSheet_1.docx]

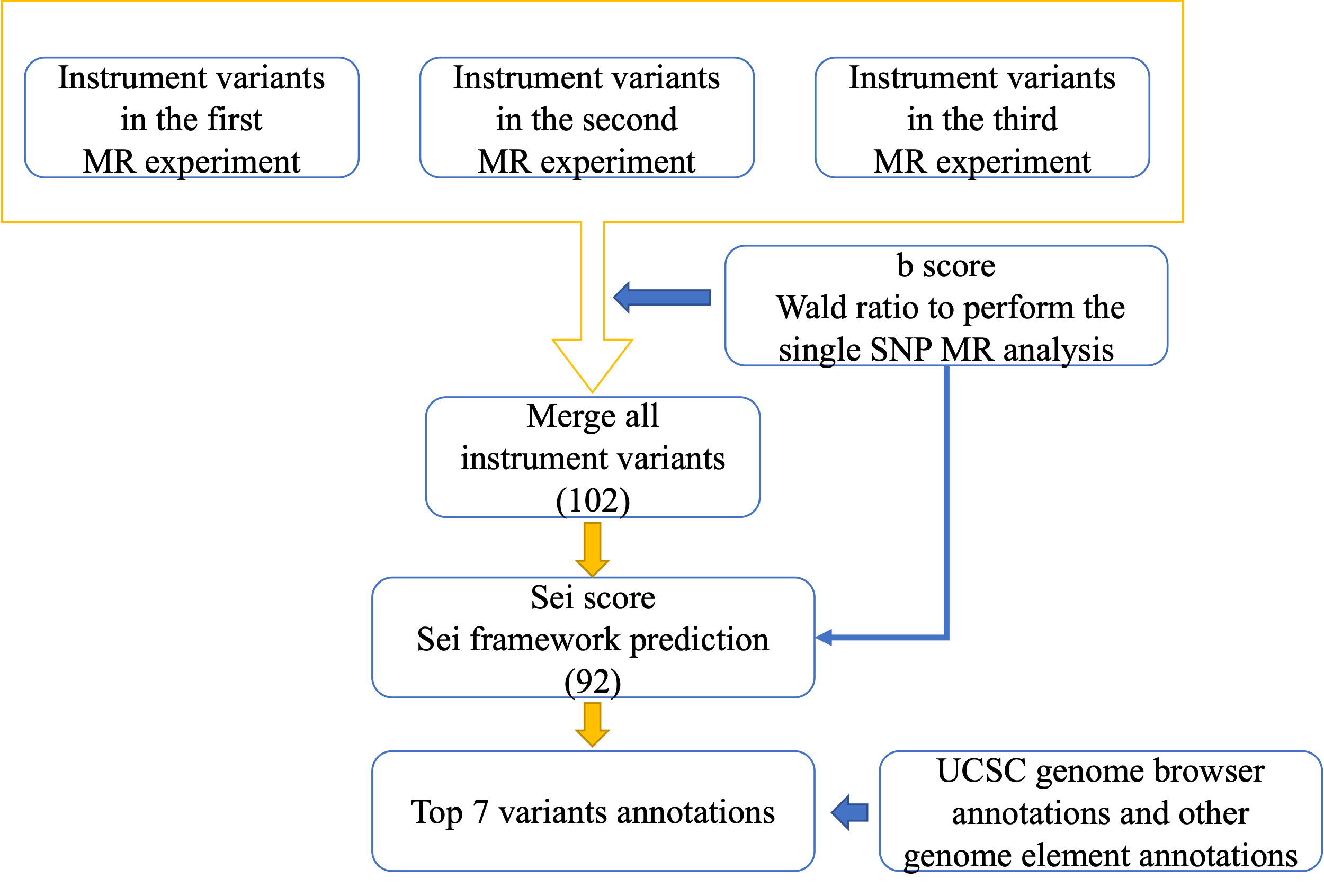


**Figure S1.** Functional annotation framework of instrument variants.

The instrument variants as the SNPs in three MR experiments and merged as the 102 unique variants to annotate. Wald ratio test was adopted to perform the single SNP MR analysis and got the b score. Sei framework was used to get the Sei score. The top 7 variants with absolute Sei score > 1 and b core > 0.5 were selected to make detailed genome element annotations. The specific annotation codes were in <https://github.com/shify-nxu/MR-RA/tree/main/Hypothyroidism_RA_script> and <https://github.com/shify-nxu/MR-RA/tree/main/Annotation_plot>.

**
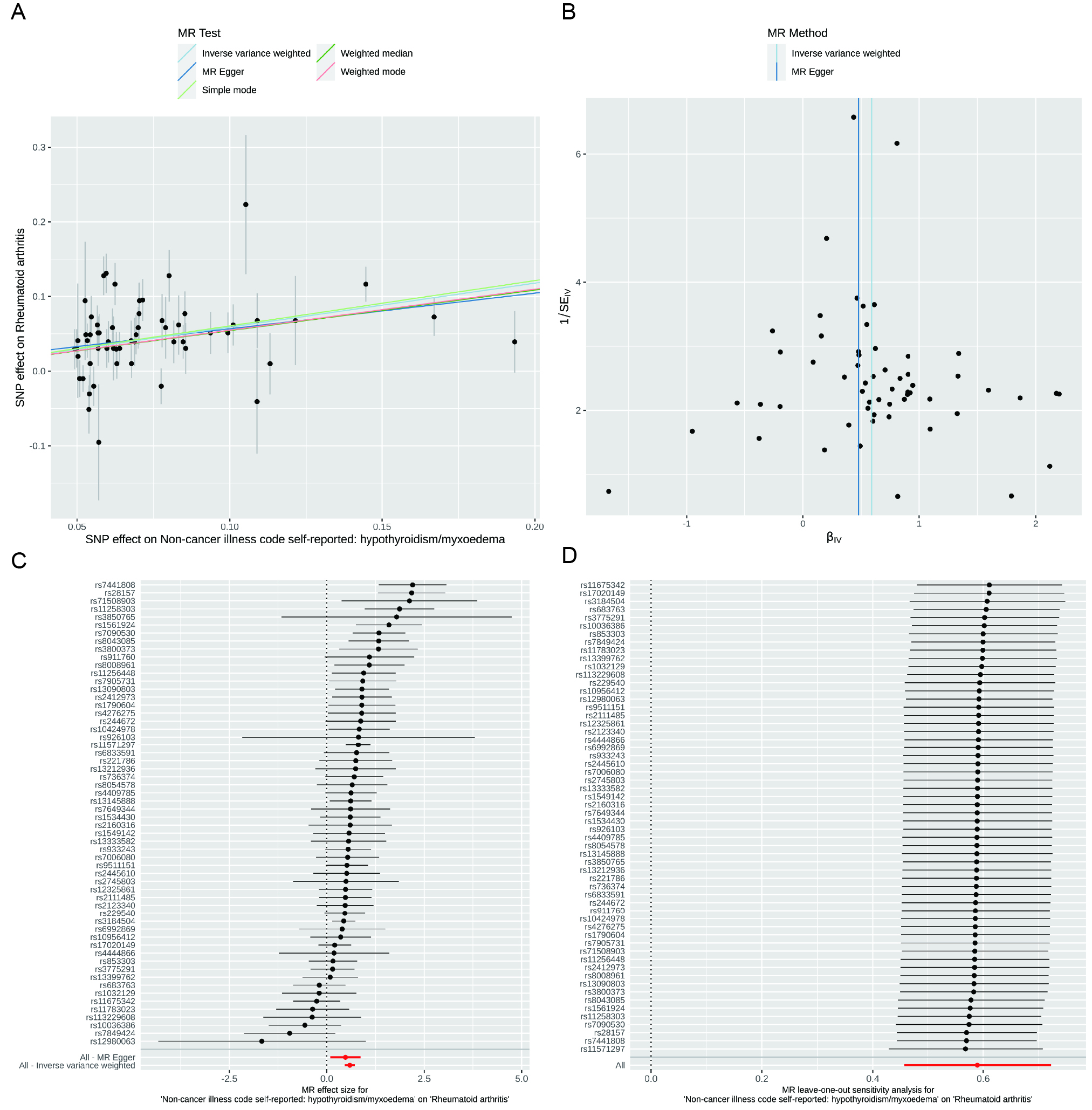
**

**Figure S2.** SNP effect evaluation in TSMR between self-reported: hypothyroidism/myxoedema and RA.

**A** MR test scatter plot of five methods. The x-axis is the SNP effect on self-reported: hypothyroidism/myxoedema. The y-axis is the SNP effect on RA. **B** MR funnel plot of IVW and MR Egger methods. **C** Forest plot of MR sensitivity analysis. All-MR Egger and IVM methods showed that MR effect sizes are larger than 0 means that self-reported: hypothyroidism/myxoedema had causal effect on RA. **D** Forest plot of MR leave-one-out sensitivity analysis.

**
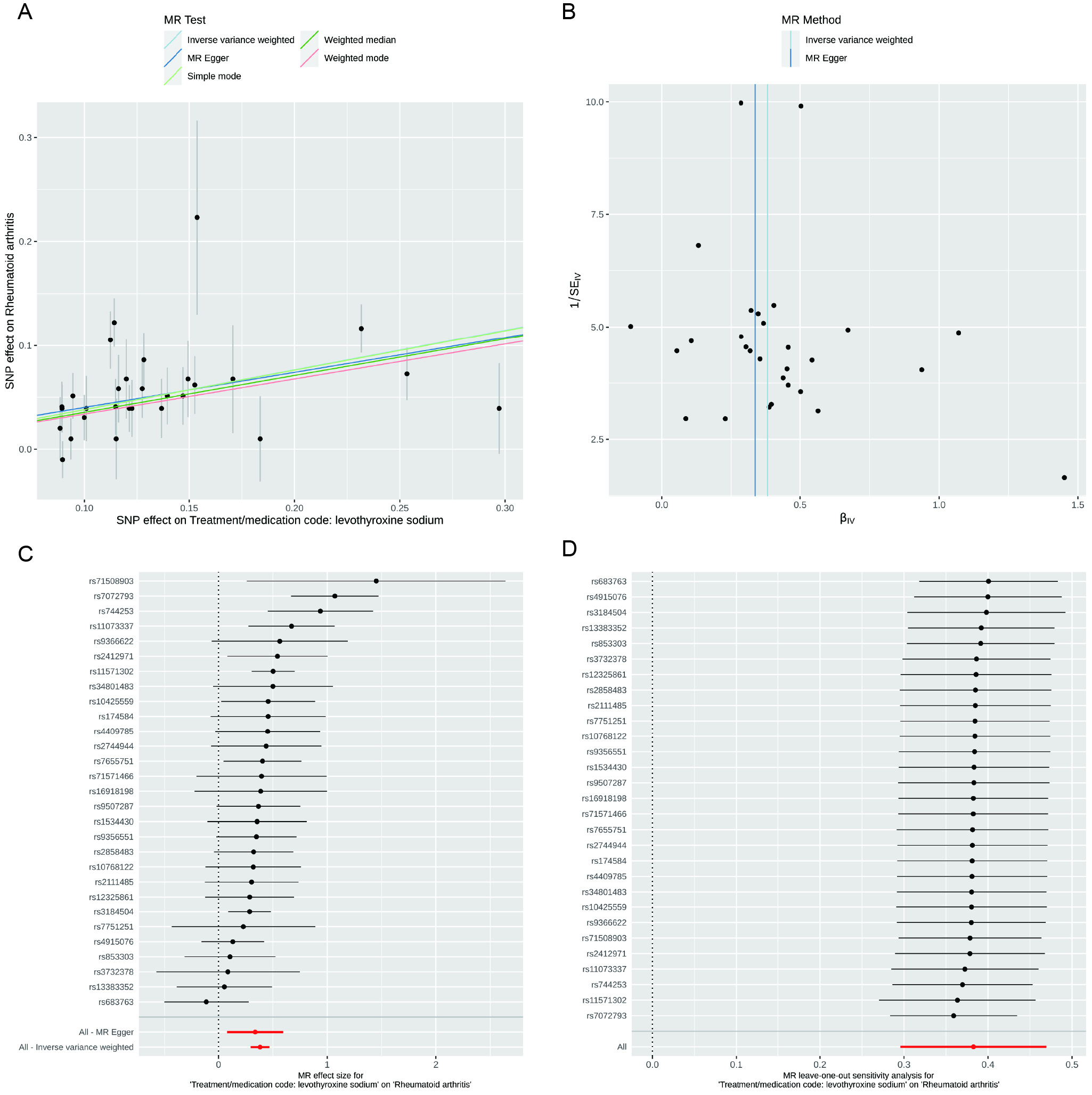
**

**Figure S3.** SNP effect evaluation in TSMR between levothyroxine sodium and RA.

**A** MR test scatter plot of five methods. The x-axis is the SNP effect on levothyroxine sodium and the y-axis is the SNP effect on rheumatoid arthritis. **B** MR funnel plot of IVW and MR Egger methods. **C** Forest plot of MR sensitivity analysis. All-MR Egger and IVM methods showed that MR effect sizes are larger than 0 means that levothyroxine sodium had causal effect on RA. **D** Forest plot of MR leave-one-out sensitivity analysis.

**
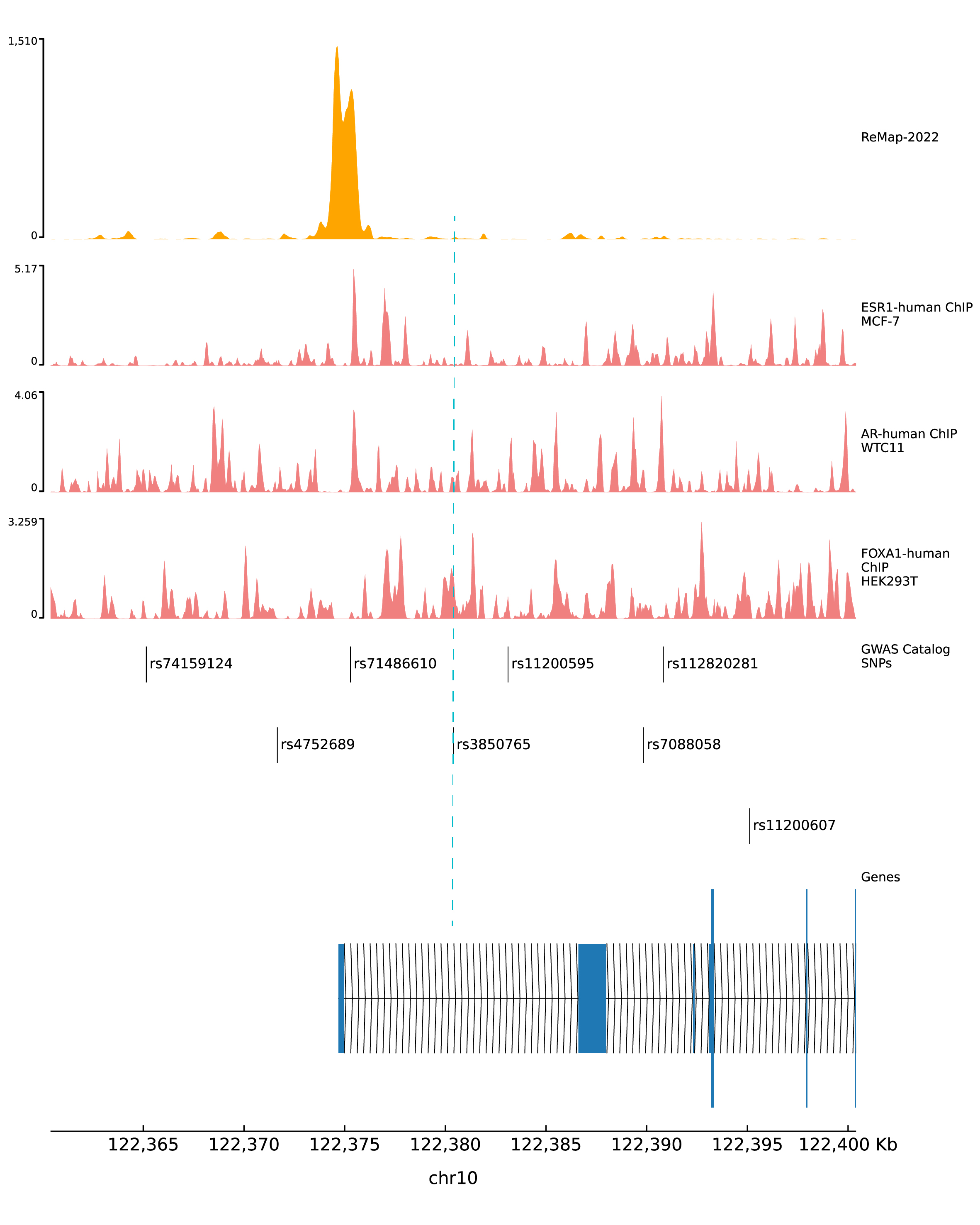
**

**Figure S4.** Functional annotations of SNP rs3850765.

The top 1 track is the density track of ReMap 2022. The top 2 track is the ChIP-seq of ESR1 in MCF-7 cell, the top 3 track is the ChIP-seq of AR in WTC11 cell and the top 4 track is the ChIP-seq of FOXA1 in HEK293T cell.

**Figure S5.** UCSC genome annotations of rs3850765.

**Figure S6.** UCSC genome annotations of rs4409785.


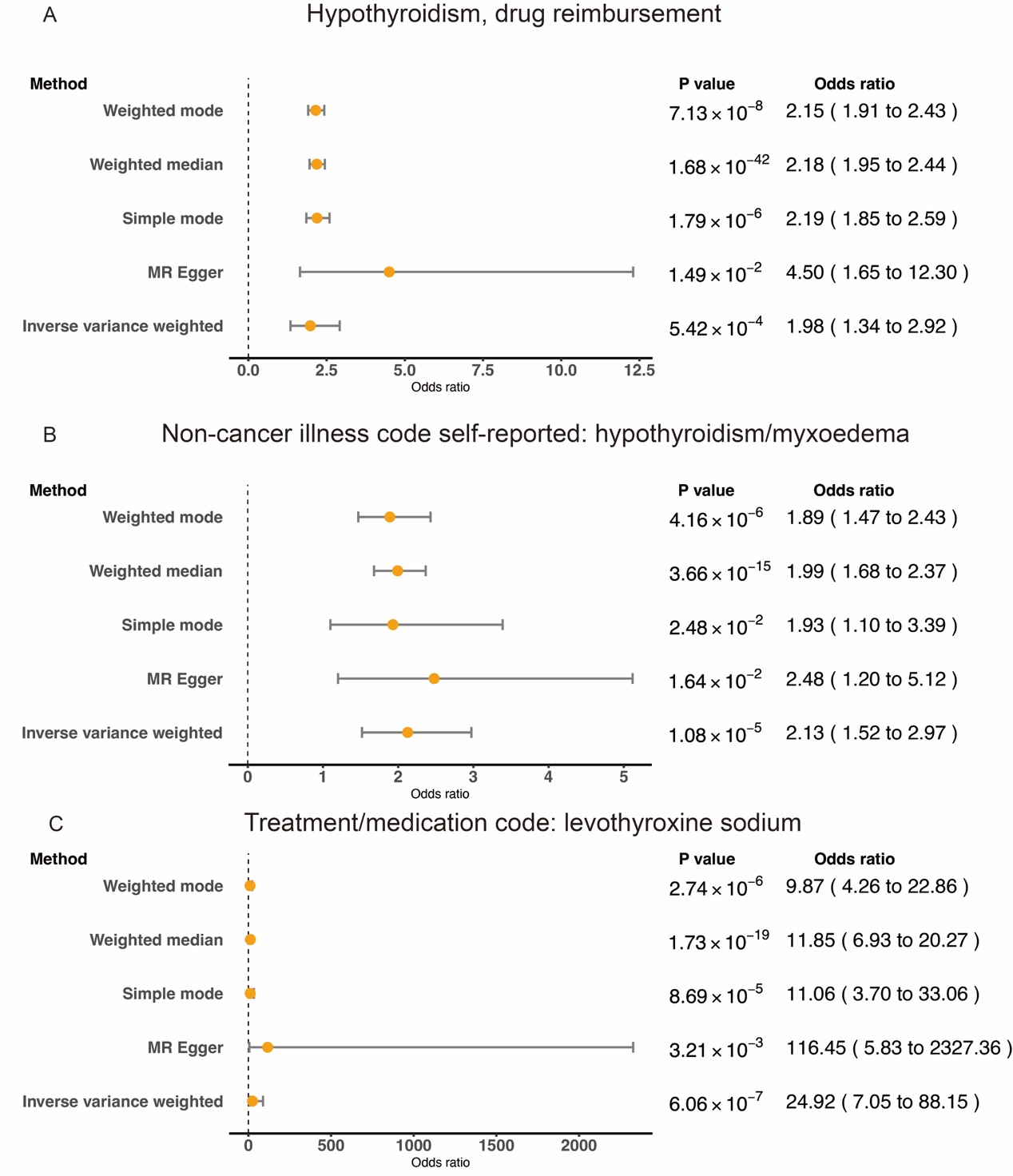


**Figure S7** Summary view of the MR results on larger datasets.

Summary of MR analysis results derived from the inverse variance weighted, MR Egger, simple mode, weighted mode and weighted mean methods. RA was used as the outcome. The hypothyroidism and drug reimbursements **(A)** non-cancer illness code self-reported: hypothyroidism/myxoedema. **(B)** and levothyroxine sodium **(C)** as the exposures and significant associations were detected for these traits and RA risk.

**
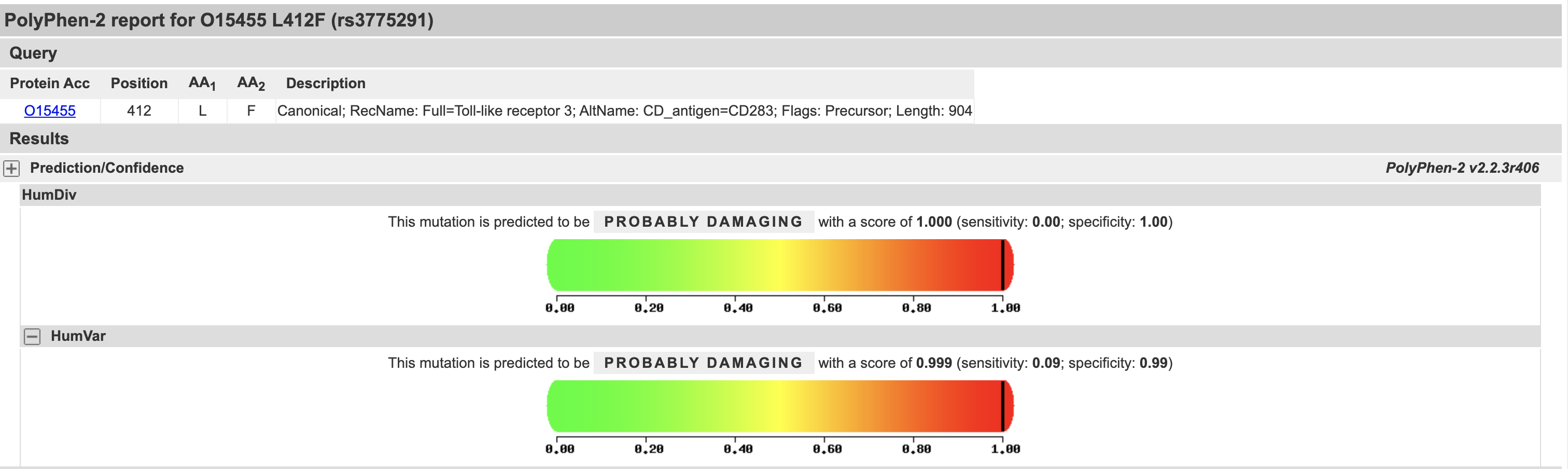
**

**Figure S8.** PolyPhen-2 pinpoint coding variant.

PolyPhen-2 was applied to predict the SNP rs3775291 effect as probably damaging with HumDiv and HumVar models.
